# High-coverage sequencing and annotated assemblies of the budgerigar genome

**DOI:** 10.1186/2047-217X-3-11

**Published:** 2014-07-08

**Authors:** Ganeshkumar Ganapathy, Jason T Howard, James M Ward, Jianwen Li, Bo Li, Yingrui Li, Yingqi Xiong, Yong Zhang, Shiguo Zhou, David C Schwartz, Michael Schatz, Robert Aboukhalil, Olivier Fedrigo, Lisa Bukovnik, Ty Wang, Greg Wray, Isabelle Rasolonjatovo, Roger Winer, James R Knight, Sergey Koren, Wesley C Warren, Guojie Zhang, Adam M Phillippy, Erich D Jarvis

**Affiliations:** 1 Department of Neurobiology, Duke University Medical Center, Durham, NC 27710, USA; 2 National Institute of Environmental Health Sciences (NIEHS), National Institutes of Health, Research Triangle Park, Raleigh, NC 27709, USA; 3 China National Genebank, BGI-Shenzhen, Shenzhen 518083, China; 4 Department of Chemistry, The Laboratory for Molecular and Computational Genomics, Laboratory of Genetics and Biotechnology Center, University of Wisconsin, Madison, WI 53706, USA; 5 Cold Spring Harbor Laboratory, Cold Spring Harbor, New York, NY 11724, USA; 6 Institute for Genome Sciences & Policy, Duke University, Durham, NC 27710, USA; 7 Department of Biology, Center for Systems Biology, Duke University, Durham, NC 27710, USA; 8 Illumina Cambridge Ltd, Cambridge, UK; 9 454 Life Sciences, Branford, Connecticut 06405, USA; 10 Center for Bioinformatics and Computational Biology, University of Maryland, College Park, MD 20740, USA; 11 The Genome Institute, Washington University School of Medicine, St. Louis, MO 63110, USA; 12 National Biodefense Analysis and Countermeasures Center, Frederick, MD 21702, USA; 13 Advanced Liquid Logic Morrisville, Morrisville, NC 27560, USA

**Keywords:** *Melopsittacus undulatus*, Budgerigar, Parakeet, Next-generation sequencing, Hybrid assemblies, Optical maps, Vocal learning

## Abstract

**Background:**

Parrots belong to a group of behaviorally advanced vertebrates and have an advanced ability of vocal learning relative to other vocal-learning birds. They can imitate human speech, synchronize their body movements to a rhythmic beat, and understand complex concepts of referential meaning to sounds. However, little is known about the genetics of these traits. Elucidating the genetic bases would require whole genome sequencing and a robust assembly of a parrot genome.

**Findings:**

We present a genomic resource for the budgerigar, an Australian Parakeet (*Melopsittacus undulatus*) -- the most widely studied parrot species in neuroscience and behavior. We present genomic sequence data that includes over 300× raw read coverage from multiple sequencing technologies and chromosome optical maps from a single male animal. The reads and optical maps were used to create three hybrid assemblies representing some of the largest genomic scaffolds to date for a bird; two of which were annotated based on similarities to reference sets of non-redundant human, zebra finch and chicken proteins, and budgerigar transcriptome sequence assemblies. The sequence reads for this project were in part generated and used for both the Assemblathon 2 competition and the first *de novo* assembly of a giga-scale vertebrate genome utilizing PacBio single-molecule sequencing.

**Conclusions:**

Across several quality metrics, these budgerigar assemblies are comparable to or better than the chicken and zebra finch genome assemblies built from traditional Sanger sequencing reads, and are sufficient to analyze regions that are difficult to sequence and assemble, including those not yet assembled in prior bird genomes, and promoter regions of genes differentially regulated in vocal learning brain regions. This work provides valuable data and material for genome technology development and for investigating the genomics of complex behavioral traits.

## Data description

### Raw genome DNA sequence reads

**Figure 1:**
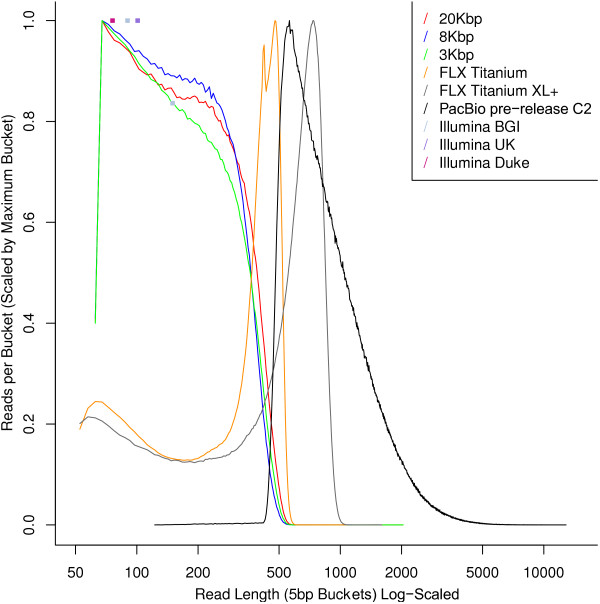
**The distribution of read lengths in 454**, **Illumina and PacBio budgerigar sequences.** The reads are binned into 5 bp buckets based on their lengths, and the fraction of reads (normalized by the size of the largest bucket) falling into each bucket is shown. Thus, curves shifted towards the right indicate longer read lengths. The reads labeled “20 Kbp”, “8 Kbp” and “3 Kbp”, “FLX Titanium” and “FLX Titanium XL+” are 454 reads. The reads labeled “PacBio pre-release C2” are uncorrected PacBio reads. The Illumina read lengths appear as colored square boxes, since these read lengths are uniform. The “Illumina Duke” reads are of length 76, The “Illumina UK” reads are of length 101, and the “Illlumina BGI” reads are of lengths 90 or 150. The longest reads come from PacBio sequencing, followed by 454 FLX + (i.e., FLX Titanium XL+) sequencing.

DNA samples were obtained from a blood sample taken from a single male *Melopsittacus undulatus*, who we aptly named Mr. B. For Illumina sequencing, reads were generated at Duke University (16×), Illumina UK (54×), and BGI (219×) using Illumina’s TruSeq [1] version2 or version3 chemistries (Table 1 and GigaDB [2]). The version3 chemistry reads through GC-rich regions, which are often found in promoters, more evenly than does version2 [3]. The insert sizes for the BGI libraries ranged from 220 bp to 40 Kbp, and the insert sizes for the Duke libraries ranged from 400–600 bp, in order to assist assemblies. Fragment sizes for the mate pair libraries, based on genome mapping, and the per base sequence quality distribution for the libraries are shown in GigaDB [2]. The Duke University Illumina libraries were sequenced at two different cluster densities: 8× coverage reads at the normal 420 k clusters/mm density and 8× coverage at a lower 350 k clusters/mm. The lower cluster density was used to increase the number of GC-rich regions sequenced. For PacBio sequencing, 6.76 Gbp (~5.5× coverage) of PacBio RS reads [4] were generated at Pacific Biosciences from two insert size libraries (7.5 K bp at 1.93× and 13 Kbp at 3.56×; PacBio reads error-corrected with Illumina can be downloaded from the supplementary webpage associated with [5]). With all reads combined, the total coverage exceeds 300× (assuming a haploid genome size of 1.23 Gbp) (Table 1), perhaps making Mr. B one of the most sequenced individual vertebrate animals as of to date. The read length distributions of these different types of reads are shown in Figure 1.

**Table 1:** Summary of genomic reads

	**Library sizes**	**Total reads**	**Total BP (Mb)**	**Coverage (assuming 1.23 Gbp genome size)**
**454**	Shotgun, 3 kb, 8 kb, 20 kb mate pair	41,898,557	19,736	15.4×
**Illumina**	220, 230, 500, 400–600, 800, 2 kb, 5 kb, 10 kb, 20 kb, 40 kb paired end	561,074,047	356,597	289×
**Pacific Biosciences**	7.5Kb, 13 kb	4,176,242	6,763	5.5×
**Combined**		607,148,846	383,096	309.9×

### Fosmid Library

To validate the assemblies in the Assemblathon 2 competition, a fosmid library was created from sheared genomic DNA (35–40 Kbp) of Mr. B [6]. Ten pools of clones were generated and sequenced using Illumina as described in [7]. Each pool of reads was individually assembled using Velvet [8]. The fosmid assemblies have been deposited at GigaDB [2]).

### Transcriptome Reads

454 FLX transcriptome reads were generated from brain RNA isolated from two males, neither of whom was Mr. B. An initial set of sequencing runs of both males was conducted at Washington University at St. Louis, producing 89.2 Mb of transcriptome sequence as reported in [9] (NCBI accession numbers SRR029329–30) and were assembled using Newbler [10] into 19,198 contigs. An additional 21× coverage (run label GK0K2XF01) was generated at Duke University from one of the males.

### Assemblies

We present three hybrid assemblies: 1) Budgerigar 454-illumina hybrid v6.3 using the CABOG assembler; 2) Budgerigar PBcR hybrid using the CABOG assembler; and 3) Budgerigar illumina-454 hybrid using the SOAPdenovo*2* assembler. The first two assemblies were annotated, after which, optical-map assisted megascaffolds were constructed based on them. As of yet, the SOAPdenovo*2* assemblies have not been annotated or aligned to optical maps. The quality statistics of these assemblies are in listed in Table 2, and brief descriptions of their construction and relative quality are provided in Additional file 1.

**Table 2:** Summary of assemblies

	**Budgerigar**_**v6.3**	**PBcR**	**Megascaffolds from Budgerigar_v6.3 + Optical Map**	**Megascaffolds from PBcR + Optical Map**	**Illumina + 454 SOAPdenovo** ***2***	**Zebra Finch **[15]	**Chicken *v4 *** [13]^*^	**Chicken *v3 *** [16]	**Peregrine Falcon **[17]	**Puerto Rican Parrot **[21]	**Macaw 1.1 **[20]
Assembler	Celera CABOG [25]	PBcR assembler [5]			SOAPdenovo*2*[26]	PCAP [27]	NA	PCAP [27]	SOAPdenovo [28,29]	Ray [30]	CLC Genomics Workbench
Sequence method	454 FLX, FLX+, Illumina	PacBio corrected with Illumina, 454 FL×, FL×+	454 FLX, FLX+, Illumina, Optical Maps.	PacBio corrected with Illumina, 454 FL×, FL×+, Optical Maps.	Illumina, 454 FL×+	Sanger	Sanger, 454	Sanger v2.1	Illumina	Illumina	Illumina, 454 FL×+
Coverage	14×	17×			137.59 Illumina, 6.85 FL×+	6×	19.1×	7.1×s	107×	26.9×	26×
Genome size	1.2Gbp	1.2Gbp	1.2Gbp	1.2Gbp	1.2Gbp	1.2Gbp	1.2Gbp	1.05Gbp	1.2Gbp	1.58Gbp	1.2 Gbp
Total bases in scaffolds	1,117,358,947	1,219,132,003	1,118,758,630	1,241,439,339	1,169,860,945	1,224,525,252	1,046,932,099	1,047,124,295	1,174,046,505	1,164,566,833	997,000
Number of scaffolds	25,212	54,668	25,163	54,138	151,393	37,698	15,932	23,776	21,224	148,255	140,453
Avg. scaffold size	44,319	22,300	44,460	22,931	7,727	32,482	65,713	44,041	55,317	7,855	Not available
N50 scaffold size	10,614,387	1,705,751	13,823,040	7,280,340	13,497,021	10,409,499	90,216,835	11,125,310	3,891,469	19,470	15,968
Largest scaffold size	39,887,647	11,564,683	61,483,320	33,208,800	66,566,439	56,620,707	195,276,750	51,053,708	18,327,016	206,462	177,843
Total gaps in scaffolds	51,150	26,444	51,295^#^	27,118	60810	124,736	NA	NA	77,368	Not available	Not available
Number of Contigs	70,863	77,556	NA	NA	212,203	126,053	27,027	85,191	98,540	259,423	214,754^*^
Avg. contig size	15,334	15,344	NA	NA	4664	9,714	38,736	12,291	11,914	4,304	Not available
N50 contig size	55,633	102,885	NA	NA	51,034	38,549	279,750	45,280	28,599	6,983	6,366
Largest contig size	465,633	849,044	NA	NA	500,974	424,635	NA	624,663	247,807	75,003	87,225

*The Chicken v4 assembly consists of chromosomes and not scaffolds with explains the very high scaffold length statistics.

^#^The increased number of gaps in megascaffolds reflects the fact that each megascaffold may be merger of many original scaffolds with gaps in between them.

### Validating sequence assemblies with optical maps

Optical Mapping is a single molecule system for the construction of ordered restriction maps of whole genomes [11], and it has been used to guide and validate sequence assemblies [12]. An optical map for the budgerirgar genome was created, using a method described in Additional file 1. The optical map contigs ranged in size from 2 Mbp to 74 Mbp and spanned over 900 Mbp with a resolution of 13.94 Kbp (i.e., one non-redundant SwaI every 13.94 Kbp). The contigs were then aligned to *in silico* restriction maps generated from Budgerigar_v6.3 and PBcR assembly scaffolds in order to validate the scaffolds. An approximate 859.21 Mb of the optical maps aligned to the Budgerigar_v6.3 assembly, in 146 scaffolds with 3 or more SwaI restriction fragments (excluding ends and fragments less than 0.4 Kbp). Of these 146 scaffolds, 43 appeared chimeric (i.e., aligned to two or more optical map contigs). For the PBcR assembly, 796.63 Mbp optical map contigs aligned, in 673 scaffolds. Of the 673 scaffolds, only 51 were chimeric. Thus, while the Budgerigar_v6.3 assembly has a higher N50 scaffold metric and hence longer scaffolds compared to the PBcR assembly, 30% the v6.3 scaffolds are chimeric, whereas only 7.6% of the PBcR assembly are chimeric.

### Optical map assisted assemblies

We took both Budgerigar_v6.3 and PBcR assemblies and filtered out alignments that did not extend to the end of either the genomic sequence scaffold or the optical map. The remaining high-quality alignments were then used to identify optical map alignments that bridged scaffolds, such that a single optical map aligned to the ends of at least two sequence scaffolds. We then iteratively extended the megascaffolds beyond pairs of sequence scaffolds, using three heuristics: (1) we limited the overhangs (i.e., the portion of the scaffold sequence that does not align to the optical map) to 2 Mbp total; (2) we bridged two scaffolds together only if the size of the gap separating them is less than 2 Mbp of Ns; and (3) if a sequence scaffold aligned to more than one optical map, we placed it into the largest one it aligns with. The above procedure slightly reduced the number of scaffolds from 25,212 to 25,163 in the Budgerigar_v6.3 assembly, and from 54,668 to 54,138 in the PBcR assembly. This relatively small change in number is expected as our procedure tended to join only sequence scaffolds that were already fairly large into even larger megascaffolds, since it is only possible to confidently align an optical map to a fairly large sequence scaffold bearing numerous SwaI restriction sites. However, this analysis substantially improved the scaffold N50 sizes from 10.6 Mbp to 13.8 Mbp in the Budgerigar_v6.3, and 1.7 Mbp to 7.3 Mbp in the PBcR assemblies, respectively (Table 2). Without limiting the length of the overhangs and gap sizes to 2 Mbp, the increase in N50 scaffold sizes in the Budgerigar_v6.3 is 17.1 Mbp (which we think could be an artifact). We speculate that some of the large gaps in the optical map correspond to centromeres or highly repetitive DNA that are difficult to assemble.

### Annotations

The Budgerigar_v6.3 and PBcR assemblies were annotated at BGI for protein coding genes by first generating a reference set of human, chicken and zebra finch proteins, and then aligning the reference set to the assemblies, and propagating annotations to 30% coverage of the reference at TBlastN, E = 1e^−5^. For the Budgerigar_v6.3 assembly, the reference set comprised of human proteins from Ensembl 60 and a set of zebra finch and chicken proteins re-annotated based on these human proteins, using a custom BGI pipeline reported on separately (Jarvis *et al*. in preparation; Zhang *et al*., in preparation). For the PBcR assembly, the reference set comprised of the Ensembl 60 human, chicken and zebra finch proteins. The propagation of these reference sets to the budgerigar assemblies is described in more detail in Additional file 1. Further, in the PBcR assembly, UTRs were annotated for 6,203 genes using the GK0K2XF01 transcriptome runs with a pipeline similar to the one described in [13]. The assembly annotations were then propagated to the corresponding sets of megascaffolds. No *de novo* gene annotations were performed.

The annotated Budgerigar assemblies had fewer genes (15,470 and 16,204 genes in the Budgerigar_v6.3 and PBcR assemblies respectively) than the published Zebra Finch (18,618 genes) and Chicken genome assemblies (17,108 genes in the 2011 Galgal4 assembly [14]). We believe the lower number of annotated genes in budgerigar assemblies is due to the differences in annotation methods rather than assembly completeness, for two reasons: (1) These annotations were produced based on similarities to zebra finch, chicken and human proteins, and hence they cannot contain more genes than the source genome annotations; and (2) The independent GenScan annotation of the Budgerigar_v6.3 assembly at the UCSC Genome Browser contains more genes than in zebra finch and chicken, 24,095 in total.

### Comparisons to other avian assemblies

**Figure 2:**
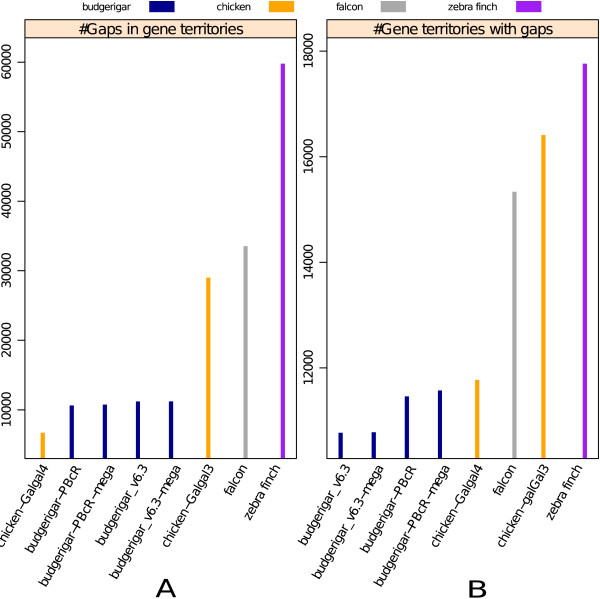
**Number of nucleotide gaps assess relative assembly incompleteness. A)** Shows the total number of gaps in genes and the surrounding 10,000 base pair regions upstream and downstream (collectively called gene territories). **B)** Shows the number of such gene territories with gaps. In both the panels, different species assemblies are colored differently, with the budgerigar assemblies shown in dark blue. The budgerigar assemblies with the “-mega” suffix are optical map enhanced versions of the Budgerigar_v6.3 and PBcR assemblies. The budgerigar assemblies have the highest numbers of gapless gene territories (right panel) and the fewest number of gaps of all assemblies except the recent chicken v4 assembly, which used a similar technology (left panel).

Our budgerigar genome assemblies were compared with the zebra finch, chicken, and falcon genomes [15-17]. The other assemblies from the Assemblathon 2 competition are available from GigaDB [18]. The zebra finch and chicken had similar contig and scaffold N50 values (38.5 kb and 10.4 Mb for zebra finch, and 279.8 kb and 90.2 Mb for chicken, respectively). In addition, since the Peregrine Falcon is the closest relative to parrots [19], we also compared the budgerigar genome assemblies to this bird. However, it was not possible to do an in depth comparison of these genomes to the recently sequenced Scarlet Macaw and Puerto Rican Parrot genomes [20,21], because both bird genomes had N50 scaffold sizes under 20,000 and N50 contig sizes under 7,000. A summary of assemblies, including the Scarlet Macaw and Puerto Rican Parrot, are shown in Table 2. Apart from the standard genome assembly quality statistics, we assessed the quality of the budgerigar assemblies along two other dimensions: (1) the coverage of highly conserved avian exons, and (2) the number of gaps 10 Kbp upstream and downstream of each gene (gene territories), and conversely, the number gene territories assembled without gaps. Of 3,288 highly conserved exons (>86% coverage across >87% of their length) we identified between chicken and zebra finch, 3,165 (96.25%) and 3,134 (95.31%) were covered with >86% identity across >87% of their length in the Budgerigar_v6.3 and PBcR assemblies respectively, pointing to good coverage of coding regions in these assemblies. The budgerigar assemblies had fewer gaps within the coding sequences and gene territories than all other avian genomes examined, except the newer unpublished Galgal4 chicken assembly that is similar to the budgerigar in that it is a hybrid that includes both short and long sequences (Sanger and 454 FLX+) (Figure 2). This suggests that our budgerigar assemblies have very well assembled genes and promoter regions.

**Figure 3:**
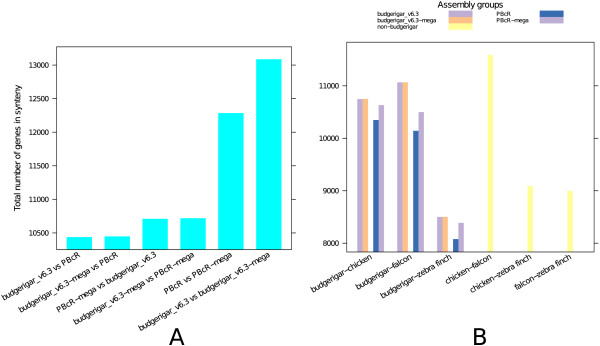
**The number of genes that are part of a syntenic block between different budgerigar assemblies (A) and between budgerigar and non**-**budgerigar assemblies (B).** The numbers were calculated from CoGE syntenic dotplots (not shown), as the total number of genes represented in syntenic blocks. The y-axis limits have been cut off close to the minimum value in the plot to show a more detailed spread of values.

Using the online CoGe tool [22-24], we assessed the structural similarities between the various budgerigar assemblies and other avian assemblies [25-30], by computing the level of coding sequence synteny among assemblies. The highest numbers of genes in synteny were observed, as expected, between a budgerigar assembly and the optical map assisted version of the same assembly (Figure 3A). However, the number of genes in synteny between the Budgerigar_v6.3 and the PBcR assemblies was similar to the number of genes in synteny between budgerigar and falcon (Figure 3A, B). Further, the number of genes in synteny did not strictly reflect phylogenetic relationships, as the zebra finch and budgerigar, close relatives [19], had a lower level of synteny than budgerigar and chicken. In addition, a number of inversions were observed even in the syntenic dotplots between the original budgerigar assemblies and their optical map-assisted assemblies (88 inversions between Budgerigar_v6.3 and Budgerigar_v6.3_mega; 209 inversions between PBcR and PBcR_mega, plots shown in GigaDB [2]). This suggests that synteny based on CoGE syntenic maps is affected by the quality of the assemblies and the characteristics of the synteny algorithm. Thus, the number of genes in synteny computed using the available methods is only a rough measure of the actual structural similarity between the assemblies compared.

In summary, this study shows that the budgerigar genomic resource we have generated has provided [5,6] (and is still expected to provide more) valuable data and material for genome technology development and for further investigating complex behavioral traits at the genomics level.

All procedures on live animals were approved by the Institutional Animal Care and Use Committee of Duke University.

## Availability and requirements

The genomic sequence reads have been deposited in NCBI’s sequence read archives (SRA) and the EBI’s ENA archive, under the same project accession number ERP002324. The SOAPdenovo*2* assembly has been submitted to GigaDB by the Assemblathon 2 team and is available at GigaDB [18]. Other supporting resources that have been deposited in GigaDB [2] are:

•Duke University brain transcriptome reads.

•Budgerigar_v6.3, PBcR assemblies (contigs and scaffolds) and optical map assisted megascaffolds based on these two assemblies (two contigs and four scaffolds in total).

•The per base sequence quality distribution of the paired end and mate paired libraries. The estimated fragment length distribution of the mate paired libraries. Peptide and coding sequences (CDS) for the Budgerigar_v6.3 and PBcR assemblies.

•Gene annotations and Repeat Masker annotations for the scaffolds.

•Optical map alignments of Budgerigar_v6.3 and PBcR assemblies in Microsoft Excel and XML formats and software (Gnomspace.rar) to view the XML alignments.

•The optical map dataset.

## Abbreviations

CABOG: Celera assembler with the best overlap graph; CoGE: Comparative genomics; PBcR: Pac bio corrected reads; XML: Extensible markup language.

## Competing interests

The authors declare that they have no competing interests.

## Authors’ contributions

JH, GG, JW, JL, BL, OF, LB, TW, GW, IR, RW, JK, WW, GZ, and EDJ contributed to generating and analyzing the genomic reads. S.K, JW, AP, MS, RA, WW, EDJ contributed to the genome assemblies. SZ, DCS, MS, RA worked on generating the optical maps and optical map assemblies. JH, JW, OF, LB, TW, GW, WW, AP, EDJ contributed to generating and analyzing the transcriptome reads. GG, JH, and EDJ wrote the paper. All authors read and approved the final manuscript.

## Authors’ information

JH, EJ, GZ are members of the Bird 10 K project.

## Supplementary Material

Additional file 1 Supplementary materials. Click here for file
