# Supplementary material for: High-coverage sequencing and annotated assemblies of the budgerigar genome
Source: Gigascience. 2014 Jul 8;3:11. doi: 10.1186/2047-217X-3-11 (PMC4109783; doi:10.1186/2047-217X-3-11)
Supplement: Additional file 1 — Supplementary materials. [file 2047-217X-3-11-S1.docx]

**Additional File 1**

**Background**

Parrots are some of the belong to a group of behaviorally advanced vertebrates groups and have an advanced ability of vocal learning relative to other vocal-learning birds. They can imitate human speech, synchronize their body movements to a rhythmic beat, and understand complex concepts of referential meaning to sounds [1–5]. However, little is known about the genetics of these traits. Elucidating the genetic bases would require whole genome sequencing and a robust assembly of a parrot genome.

**Raw genome DNA sequencing reads**

We performed several blood bleeds of the alar vein, from which we isolated high molecular weight (MW) genomic DNA using Qiagen Genomic tip 500G (Catalog number 10262). For 454 sequencing, the DNA was fragmented using a HydroShear apparatus from Genomic Solutions Ltd, prepared for shotgun (FLX and FLX+), 3Kbp, 8Kbp, and 20Kbp mate-paired (MP) library sizes as described in[6]. The 454 sequences were sequenced to a total of 15.4X coverage (Table 1).

**Assemblies**

The quality statistics of three hybrid assemblies are listed in Table 2. Brief descriptions of their construction and relative quality are provided:

1. 454-Illumina hybrid CABOG assembly (known as Budgerigar_v6.3). CABOG [7] [] was used to assemble 15.4X coverage 454 FLX (~200 bp) and FLX+ (~700 bp) reads from multiple insert size libraries (range 0.4 to 20Kb) combined with 7.8X coverage of 75bp paired-end Illumina reads. Details of the parameters for the CABOG assembler are in Additional file v6-sli. The Illumina data was primarily used for improving consensus accuracy, and the paired-end information was not used in the assembly. Instead, the paired end information of the 454 data was used for scaffolding. The N50 scaffold size is greater than those of the zebra finch and chicken galGal3 (v3) assemblies (Table 2). This assembly has also been independently annotated and posted in the [NCBI](http://www.ncbi.nlm.nih.gov/genome/10765) [8] and [UCSC Genome Browser](http://genome.ucsc.edu/cgi-bin/hgGateway?hgsid=334955181&clade=vertebrate&org=Budgerigar&db=0) [9] pages for Budgerigar. This assembly was *not* entered in Assemblathon 2, because the competition required that the assembly be not be improved with iterative feedback from other published genomes.
2. PacBio corrected reads (PBcR) hybrid assembly. First, 5.5X PacBio RS reads were error corrected with 54X Illumina reads (comprising 400-600bp paired-end Illumina reads (see GigaDB [10]) to obtain 3.75X corrected PacBio reads [11]. The PBcR reads were then combined with the 15.4X 454 reads to build a hybrid assembly using CABOG. Relative to the Budgerigar_v6.3 assembly, the N50 contig size of the PBcR assembly was greatly improved (Table 2); however, the N50 scaffold length was greatly reduced (Table 2). It appears that the combination of multiple technologies negatively affects the CABOG scaffolding module, and similar effects have been observed when combing 454 and Illumina data in hybrid CABOG assemblies. The PBcR assembly was entered in the Assemblathon 2 competition as the “CBCB” bird assembly, where it obtained the 7^th^ best cumulative assembly quality score and the 5^th^ best average rank out of 14 assemblies [12]. The assembly is available at [13] under the Assemblathon code name bird_9C.
3. Illumina-454 hybrid SOAPdenovo*2* assembly. SOAPdenovo*2* [14] was used to assemble 137.59X Illumina reads from with multiple insert size libraries (range 0.2 to 40Kb). Additionally, 6.85X 454 FLX+ data were used to improve the assembly using a custom BGI pipeline. The hybrid assembly has the highest N50 scaffold and contig sizes and the longest scaffolds and contigs of all the budgerigar assemblies presented in this paper (Table 2). However, it also has many more scaffolds and contigs, suggesting a preponderance of short scaffolds and contigs. This assembly was entered in Assemblathon 2 competition as the SOAP** bird assembly, where it obtained the 5^th^ best cumulative assembly quality score and the 4^th^ best average rank out of 14 assemblies [11]. This assembly is available at [13] under the Assemblathon code name bird_14E.

**Annotation of Budgerigar PacBio assembly based on human, chicken and zebra finch proteins**

The following is the description of the common pipeline used to propagate annotations from a reference set of human, chicken and zebra finch reference protein sets to the budgerigar assemblies. However, the reference sets were different for the Budgerigar_v6.3 and the PBcR assemblies. Please refer to the “Annotations” section of the main manuscript for more details.

(a) Rough alignment.

We aligned the non-redundant protein sequences from human, chicken, and zebra finch (the longest translations were chosen to represent each gene) to the assembly by TblastN at E-value 1e^-5^, and linked the blast hits into targeted gene fragments by genBlastA. We filtered those targeted fragments with homologous block length shorter than 30% of query proteins.

(b) Precise alignment.

We then extracted the targeted nucleotide gene fragment sequences from the genome by extending 10,000 bp at both ends of the alignment regions, included the intron regions, and did the precise alignments for the parent protein sequences to these DNA fragments by Genewise. This step outputs the precise structure of the homologous genes in the target genomes.

(c) Overlapping gene filtering.

We merged all three sets from human, chicken and finch to make a reference set. Transcripts were clustered by loci overlapping. Only the one with the longest transcript was kept in every cluster.

**Validating sequence assemblies with optical maps**

To create an optical map for the budgerigar genome, DNA from Mr. B was first stretched out into single molecules in nanotubes and cut with the enzyme SwaI, using a described protocol [15]. The individual DNA molecule restriction maps (Rmaps) were *de novo* assembled into 93 whole genome optical map contigs using the algorithm *Germinate and Grow (G&G)* [16].

**References**

1. Pepperberg IM: **Vocal learning in Grey parrots: A brief review of perception, production, and cross-species comparisons**. *Brain Lang* 2010, **115**:81–91. [*Special Issue on Language and Birdsong*]

2. Schachner A, Brady TF, Pepperberg IM, Hauser MD: **Spontaneous Motor Entrainment to Music in Multiple Vocal Mimicking Species**. *Curr Biol* 2009, **19**:831–836.

3. Petkov CI, Jarvis E: **Birds, primates, and spoken language origins: behavioral phenotypes and neurobiological substrates**. *Front Evol Neurosci* 2012, **4**:12.

4. Patel AD, Iversen JR, Bregman MR, Schulz I: **Experimental Evidence for Synchronization to a Musical Beat in a Nonhuman Animal**. *Curr Biol* 2009, **19**:827–830.

5. Jarvis ED: **Learned Birdsong and the Neurobiology of Human Language**. *Ann N Y Acad Sci* 2004, **1016**:749–777.

6. Prüfer K, Munch K, Hellmann I, Akagi K, Miller JR, Walenz B, Koren S, Sutton G, Kodira C, Winer R, Knight JR, Mullikin JC, Meader SJ, Ponting CP, Lunter G, Higashino S, Hobolth A, Dutheil J, Karakoç E, Alkan C, Sajjadian S, Catacchio CR, Ventura M, Marques-Bonet T, Eichler EE, André C, Atencia R, Mugisha L, Junhold J, Patterson N, et al.: **The bonobo genome compared with the chimpanzee and human genomes**. *Nature* 2012, **486**:527–531.

7. Miller JR, Delcher AL, Koren S, Venter E, Walenz BP, Brownley A, Johnson J, Li K, Mobarry C, Sutton G: **Aggressive assembly of pyrosequencing reads with mates**. *Bioinformatics* 2008, **24**:2818–2824.

8. **Melopsittacus undulatus 6.3 Assembly** [http://www.ncbi.nlm.nih.gov/assembly/325078]

9. **Budgerigar (Melopsittacus undulatus) Genome Browser Gateway** [http://genome.ucsc.edu/cgi-bin/hgGateway?hgsid=371530703_RGWJYlXfsWGa3X6MBuAaAiYaYIgi&clade=vertebrate&org=Budgerigar&db=0]

10. Ganapathy, G; Howard, JT; Koren, S; Phillippy, A; Zhou, S; Schwartz, D; Schatz, M; Aboukhalil, R; Ward, JM; Li, J; Li, B; Fedrigo, O; Bukovnik, L; Wang, T; Wray, G; Rasolonjatovo, I; Winer, R; Knight, J R; Warren, W; Zhang, G; Jarvis, ED (2013): **De novo high-coverage sequencing and annotated assemblies of the budgerigar genome**. *GigaScience* Database. http://gigadb.org/dataset/100059

11. Koren S, Schatz MC, Walenz BP, Martin J, Howard JT, Ganapathy G, Wang Z, Rasko DA, McCombie WR, Jarvis ED, Phillippy AM: **Hybrid error correction and de novo assembly of single-molecule sequencing reads**. *Nat Biotechnol* 2012, **30**:693–700.

12. Bradnam KR, Fass JN, Alexandrov A, Baranay P, Bechner M, Birol I, Boisvert S, Chapman JA, Chapuis G, Chikhi R, Chitsaz H, Chou W-C, Corbeil J, Del Fabbro C, Docking TR, Durbin R, Earl D, Emrich S, Fedotov P, Fonseca NA, Ganapathy G, Gibbs RA, Gnerre S, Godzaridis E, Goldstein S, Haimel M, Hall G, Haussler D, Hiatt JB, Ho IY, et al.: **Assemblathon 2: evaluating de novo methods of genome assembly in three vertebrate species**. *GigaScience* 2013, **2**:10.

13. Bradnam, KR; Fass, JN; Alexandrov, A; Baranay, P; Bechner, M; Birol, I; Boisvert, S; Chapman, JA; Chapuis, G; Chikhi, R; Chitsaz, H; Corbeil, J; Del Fabbro, C; Docking, TR; Durbin, R; Earl, D; Emrich, S; Fedotov, P; Fonseca, NA; Ganapathy, G; Gibbs, RA; Gnerre, S; Godzaridis, É; Goldstein, S; Haimel, M; Hall, G; Haussler, D; Hiatt, JB; Ho, IY; Howard, J; Hunt, M; Jackman, SD; Jaffe, DB; Jarvis, E; Jiang, H; Kazakov, S; Kersey, PJ; Kitzman, JO; Knight, JR; Lam, T; Lavenier, D; Laviolette, F; Li, Y; Li, Z; Liu, B; Liu, Y; Luo, R; MacCallum, I; MacManes, MD; Maillet, N; Melnikov, S; Naquin, D; Ning, Z; Otto, TD; Paten, B; Paulo, OS; Phillippy, AM; Pina-Martins, F; Place, M; Przybylski, D; Qin, X; Qu, C; Ribeiro, FJ; Richards, S; Rokhsar, DS; Ruby, JG; Scalabrin, S; Schatz, MC; Schwartz, DC; Sergushichev, A; Sharpe, T; Shaw, TI; Shendure, J; Shi, Y; Simpson, JT; Song, H; Tsarev, F; Vezzi, F; Vicedomini, R; Vieira, BM; Wang, J; Worley, KC; Yin, S; Yiu, S; Yuan, J; Zhang, G; Zhang, H; Zhou, S; Korf, IF (2013): **Assemblathon 2 assemblies.** GigaScience Database. http://dx.doi.org/10.5524/100060

14. Luo R, Liu B, Xie Y, Li Z, Huang W, Yuan J, He G, Chen Y, Pan Q, Liu Y, Tang J, Wu G, Zhang H, Shi Y, Liu Y, Yu C, Wang B, Lu Y, Han C, Cheung DW, Yiu S-M, Peng S, Xiaoqian Z, Liu G, Liao X, Li Y, Yang H, Wang J, Lam T-W, Wang J: **SOAPdenovo2: an empirically improved memory-efficient short-read de novo assembler**. *GigaScience* 2012, **1**:18.

15. Dimalanta ET, Lim A, Runnheim R, Lamers C, Churas C, Forrest DK, de Pablo JJ, Graham MD, Coppersmith SN, Goldstein S, Schwartz DC: **A Microfluidic System for Large DNA Molecule Arrays**. *Anal Chem* 2004, **76**:5293–5301.

16. **Germinate & Grow: A de Bruijn assembly algorithm for optical maps - F1000Posters** [http://f1000.com/posters/browse/summary/1047].
